# A Rare Case of Neck Swelling: Acute Suppurative Thyroiditis With End-Stage Renal Disease

**DOI:** 10.7759/cureus.30044

**Published:** 2022-10-07

**Authors:** Nidhi Kaeley, Archana Bairwa, Bhoomika Kaushik, Salva A M S, Jewel Rani Jose

**Affiliations:** 1 Department of Emergency Medicine, All India Institute of Medical Sciences, Rishikesh, IND; 2 Department of Pathology and Laboratory Medicine, All India Institute of Medical Sciences, Rishikesh, IND

**Keywords:** subacute suppurative thyroiditis, fine needle aspiration cytology, thyroid abscess, thyrotoxicosis, acute suppurative thyroiditis

## Abstract

Acute suppurative thyroiditis is a rare life-threatening endocrine emergency. The thyroid gland has rich vascularity and lymphatic drainage, has large amounts of iodine in the tissue, generates hydrogen peroxide, and is encapsulated. Owing to these factors, infection of the thyroid gland is rare. The clinical presentation of acute suppurative thyroiditis closely resembles that of subacute thyroiditis, with a differentiation possible only on fine needle aspiration cytology (FNAC). However, differentiating these two conditions is important because the management of these two conditions differs drastically. Management includes intravenous antibiotics, drainage of abscesses, and sometimes surgery may be required. Here, we present a case of thyroid abscess caused by methicillin-resistant Staphylococcus aureus (MRSA), diagnosed using FNAC of the thyroid gland and blood culture.

## Introduction

Inflammation of the thyroid gland referred to as thyroiditis can have various causes. Of these, acute suppurative thyroiditis although rare, is an emergency associated with a high mortality rate if left untreated. Its presentation closely resembles that of subacute thyroiditis which is a common but self-remitting condition. Acute suppurative thyroiditis comprises <1% of thyroid disease. Here, we present a case of a young male with chronic kidney disease (CKD) who was ultimately diagnosed to have acute suppurative thyroiditis.

## Case presentation

A 23-year-old male, a diagnosed case of CKD stage 5 - on maintenance hemodialysis twice weekly, with a history of marijuana smoking with no other significant past medical history. The patient presented to the emergency with complaints of fever, which was intermittent, low grade, and undocumented, initially later progressed to high grade associated with chills and rigors in the last four days. The patient noticed a neck swelling 15 days back, progressively increasing in size associated with mild pain and difficulty in swallowing which was more for solids than liquids. The swelling was not associated with any discharge. He also complained of bilateral lower limb swelling and generalized myalgia for one week. He had a history of retrosternal, non-radiating chest pain for 7 days which was associated with palpitations and shortness of breath on exertion

On examination, pulse rate was 175/min which was irregularly irregular, blood pressure was 100/70 mmHg, respiratory rate was 24/min, saturation 100% with 12 liters of oxygen, the temperature was 102ºF, Glasgow coma scale (GCS) - E4V4M6, the patient was agitated. Pallor and bilateral pitting pedal edema were present on general examination. Respiratory system examination revealed bilateral basal crepitations, other system examinations were within normal limits. Local examination revealed a 10×5cm swelling on the right side of the neck, that was moving with deglutition, and tenderness present on palpation, but not associated with discharge, redness, or fluctuation.

An electrocardiogram suggested atrial fibrillation for which diltiazem bolus followed by infusion was given. Lung point-of-care ultrasound (POCUS) showed bilateral grouped B lines and pleural shreds with minimal pleural effusion the in lungs. Arterial blood gas revealed severe metabolic acidosis with a pH of 7.12 and HCO_3_ of 11.3 mmol/L. The patient was started on intravenous antibiotics (ceftriaxone, clindamycin) and steroids. Urgent hemodialysis was done for anuria and severe metabolic acidosis. He was then admitted to the intensive care unit. The patient's blood investigations revealed anemia, neutrophilic leukocytosis, thrombocytopenia, hyperbilirubinemia, and deranged kidney and thyroid function tests as shown in Table 1.

**Table 1:** Laboratory investigations HB = Hemoglobin, TLC = Total leukocyte count, DLC = Differential leukocyte count, SGOT = Serum Glutamic Oxaloacetic Transaminase, SGPT = Serum glutamic pyruvic transaminase, ALP = Alkaline phosphatase, GGT = Gamma-glutamyl transferase, TSH = Thyroid-stimulating hormone, FT = Free triiodothyronine, CRPH = C-Reactive protein, TPO = Thyroid peroxidase

	Day-1	Day- 6	Day-10	Day-15	Day-18	Day-21	Reference Range
HB (g/dl)	7.2	8.5	9.4	9.6	10.1	10	12-15 (g/dl)
TLC (cells/mm³)	22.2	18.04	31.05	32.9	30.9	31.8	3.8-5.2(cells/mm³)
DLC (%)	81/8/5/0.15	83/7/7/0.5	86/4/7.6/0.3	93/2/2.1/1.1	92/2.5/4.6/0.3	91/2.6/5.6/0.4	40-70/20-40/2-8/1-6(%)
Platelets (thousand/mm³)	108	130	80	55	60	75	150-400 (thousand/mm³)
Total Bilirubin (mg/dL)	4.7	2.17	1.5		1.3		0.3-1.2 (mg/dL)
Direct Bilirubin (mg/dL)	3.09	1.4	0.8		0.7		0-0.2 (mg/dL)
SGOT (U/L)	50	22.7	29		35		0-35 (U/L)
SGPT (U/L)	53	15	9.1		22		0-35(U/L)
ALP(U/L)	900	799	879		769		30-120 (U/L)
GGT(U/L)	173	138	55		68		0-38(U/L)
S. Albumin (g/dL)	3.2	3.4	2.6		1.8		3.5-5.2 (g/dL)
TSH (mcIU/mL)	0.02	0.20			1.2		0.35-5.5 (mcIU/mL)
FT3(pg/mL)	6.33	4.2			2.04		2.3-4.2 (pg/mL)
FT4(ng/mL)	2.2	2.1			1.3		0.89-1.76 (ng/mL)
Urea (mg/mL)	188	121	98	78	65	55	17-43 (mg/mL)
Creatinine (mg/mL)	7.4	4.5	3.2	3.5	2.6	2.4	0.55-1.02 (mg/mL)
CRPH (mg/L)	173	180	168		179		Less than 10 (mg/L)
Procalcitonin (ng/L)	>75	68			>75		Less than 0.1 (ng/L)
Anti TPO (U/mL)	42.7						Less than 60 (U/mL)

Other investigations done are shown in Table 2.

**Table 2:** Other investigations NCCT = non-contrast computerized tomography, HRCT = High-Resolution Computed Tomography, FNAC = Fine Needle Aspiration Cytology

Investigations	
Ultrasonogram of neck	Diffuse edema in strap muscles and subcutaneous tissue, thyroid gland enlarged with altered echo texture likely infective.
Ultrasound whole abdomen	Bilateral renal parenchymal disease with ascites.
Bilateral Lower Limb Doppler	Normal
Transthoracic Echocardiography	No regional wall motion abnormality, Left Ventricular ejection fraction-60%, and no vegetation
NCCT neck	Bulky and hypodense thyroid gland without significant tracheal and laryngeal compression.
HRCT thorax	Active infective lungs with bilateral ground glass opacities
Thyroid FNAC	Dense acute inflammatory cell infiltrates (neutrophil), cystic macrophages with few histiocytes and areas of necrosis, ZN stain was negative.
Blood culture	Methicillin-resistant Staphylococcus aureus.

**Figure 1:**
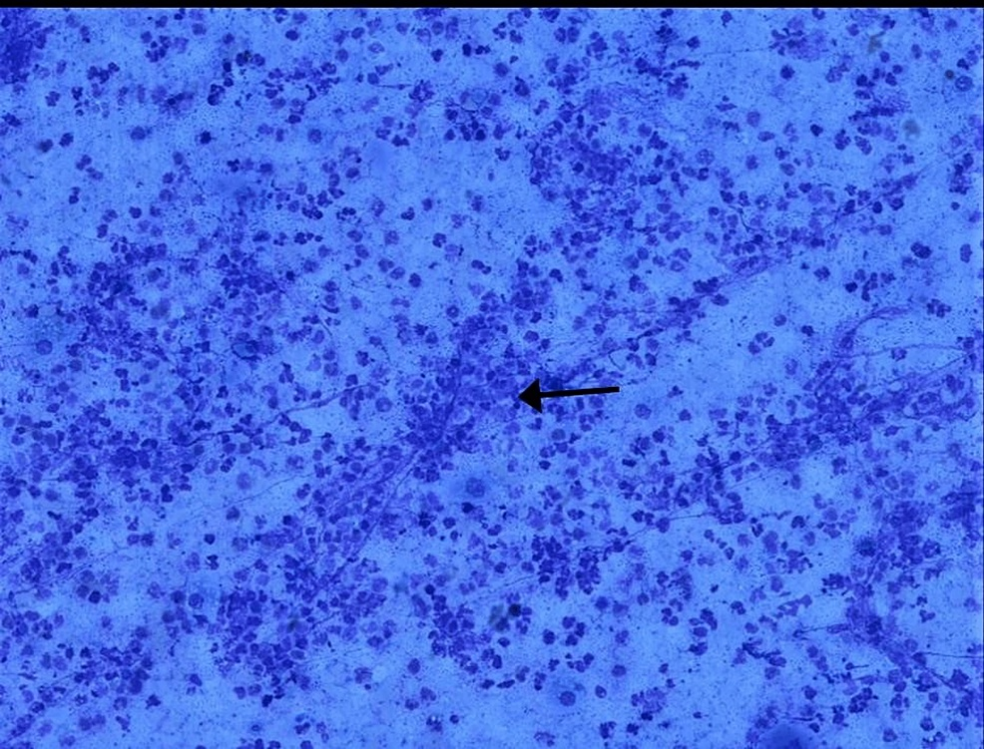
Smears show mainly dense acute inflammatory cell infiltrate (neutrophil) along with few histiocytes, lymphocytes and granulation tissue in Giemsa stain, 200x. The arrow shows acute inflammatory cell infiltrate along with granulation tissue.

**Figure 2:**
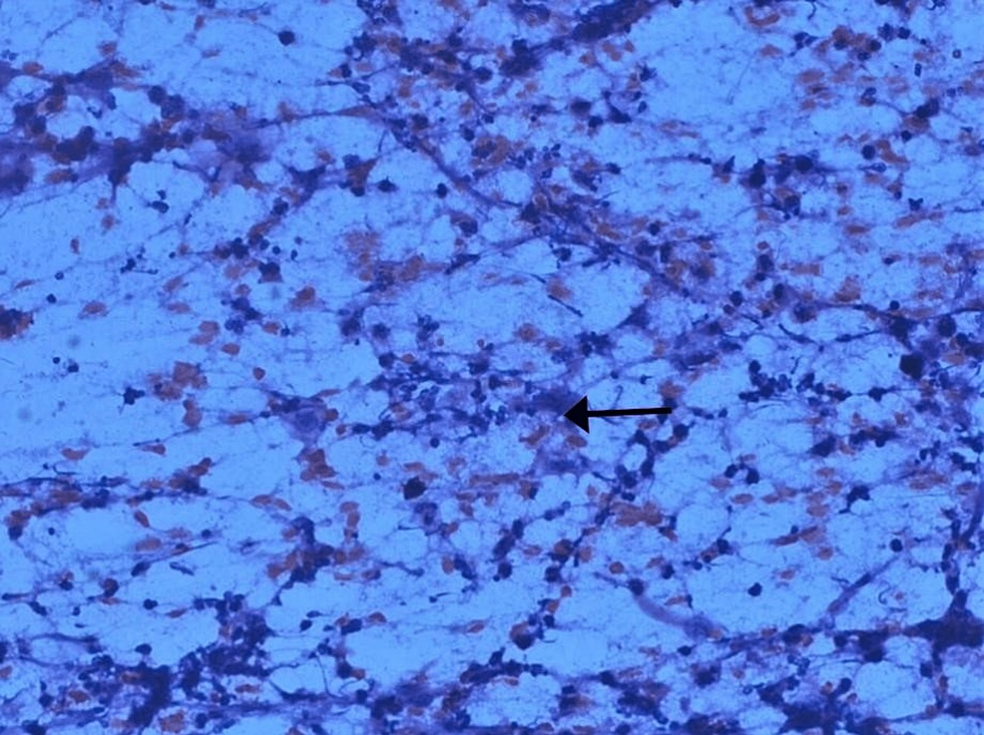
Smears showing mainly acute inflammatory cells (neutrophil) along with occasional histiocytes, lymphocytes and granulation tissue in Pap stain, 400x. The arrow shows acute inflammatory infiltrate along with granulation tissue.

The patient was started on Tab Propranolol 60 mg 6-hourly and Tab Propylthiouracil 200 mg 4-hourly, Hydrocortisone 100 mg intravenously 8-hourly. Antibiotics were stepped up to intravenous vancomycin 500 mg alternate days and meropenem 500 mg twice daily. The patient required high flow oxygen to maintain saturation; hence, it was not feasible to transport patient for thyroid scintigraphy or iodine uptake study. A thyroid fine needle aspiration cytology (FNAC) report revealed a thyroid abscess (Figures 1, 2) and the culture report did not detect any organism.

Ultrasound-guided aspiration of thyroid abscess was performed, seropurulent pus was seen and thyroid drain was inserted, and the patient improved symptomatically. A final diagnosis of acute suppurative thyroiditis and Lower respiratory tract infection (LRTI) with sepsis, acute on CKD was made. However, the patient developed severe respiratory distress with type 1 respiratory failure, and hypotension for which he was intubated, and noradrenaline infusion was started, subsequent chest imaging revealed multiple consolidations with cavitations suggestive of hospital-acquired pneumonia. Despite all efforts, the patient succumbed to death on day 22 of admission. 

## Discussion

The term thyroiditis refers to inflammation of the thyroid gland. Thyroiditis can be broadly classified into infectious, De Quervain’s, autoimmune, Riedel’s, and miscellaneous [1]. The thyroid gland has rich vascularity and lymphatic drainage, has large amounts of iodine in the tissue, generates hydrogen peroxide, and is encapsulated. Owing to these factors, infection of the thyroid gland is rare. Acute suppurative thyroiditis is a rare life-threatening endocrine emergency. It has a reported incidence of 0.1%-0.7% and if untreated, the disease carries a high mortality rate of around 12% [2,3]. On the other hand, subacute thyroiditis is a spontaneously remitting condition that is much more common than acute suppurative thyroiditis. It may last for weeks to several months and tends to recur. It is also referred to as granulomatous or De Quervain’s thyroiditis [4,5].

Multiple factors predispose patients to the development of acute suppurative thyroiditis of which immunosuppression is the most common [6]. However, pyriform sinus fistula and third and fourth branchial arch abnormalities are also important, especially in children [7,8]. The clinical features, laboratory findings, and radiological features of acute suppurative thyroiditis closely resemble those of subacute thyroiditis (Table 3).

**Table 3:** Differentiating features between acute suppurative thyroiditis and subacute suppurative thyroiditis I = iodine, F-FDG-PET =fluorodeoxyglucose positron emission tomography

Characteristic	Acute Thyroiditis	Subacute Thyroiditis
History		
Preceding Upper Respiratory Tract Infection	More common	Less common
Fever	More common	Less common
Symptoms of thyrotoxicosis	Less common	More common
Sore throat	More common	Less common
Physical Examination		
Painful thyroid swelling	More common	Less common
Left side involvement	More common	Less common
Migrating thyroid tenderness	Less common	More common
Overlying skin erythema	More common	Less common
Laboratory Investigations		
Elevated white blood cell counts	More common	Less common
Erythrocyte Sedimentation Rate > 30 mm/hr	More common	Less common
Abnormal thyroid hormone levels (Elevated or depressed)	Less common	More common
Raised alkaline phosphatase, transaminases	Rare	More common
Radiological Investigations		
^123^I uptake low	Common	~100%
Abnormal thyroid scan	More common	Less common
Gallium scan positive	~100%	~100%
^18^F-FDG-PET	Positive	Positive
Barium swallow showing fistula	More common	Less common

Fine needle aspiration from the thyroid tissue provides the most accurate diagnosis and can be used to differentiate these two conditions. In acute suppurative thyroiditis, the FNAC aspirate is purulent with the presence of bacteria or fungi, whereas, in subacute thyroiditis, the FNAC aspirate shows lymphocytes and macrophages with some giant cells [9].

Although these conditions closely mimic each other, certain clinical features can be used as differentiating factors. Patients with acute suppurative thyroiditis are generally immunocompromised, sicker, with severe localized thyroid pain, an associated upper respiratory infection, and lymphadenopathy [1]. Thyrotoxic presentation is seen in only 8%-12% of patients with bacterial thyroiditis [3]. We reviewed some recent articles on patients with acute suppurative thyroiditis, the important studies have been shown in Table 4.

**Table 4:** Details of recent articles of patients with acute suppurative thyroiditis HIV = human immunodeficiency virus, PTB = pulmonary tuberculosis, HTN = hypertension, IVDA = intravenous drug abuser, T2DM = type two diabetes mellitus, MRSA = methicillin-resistant staphylococcus aureus, MI = myocardial infarction, GERD = gastroesophageal reflux disease.

S. No	Author (Published year)	Presenting complains	Predisposing condition/Comorbidities	Organism isolated	Procedure and method	Outcome
1.	Hendricks et al. (2022) [10]	Fever, Sore throat, Odynophagia	Accidental ingestion of fish bone	-	Hemithyroidectomy, Antibiotics	Survived
2.	Siddiqui et al. (2021) [11]	Dysphagia, Dyspnea, Neck swelling with pain	HIV, PTB, HTN	Escherichia coli	Antibiotics, drainage of abscess	Survived
3.	Cheung et al. (2021) [12]	Palpitation, Atypical chest pain, fever, sore throat, Odynophagia	IVDA, T2DM HTN	MRSA	Antibiotics	Died from MI
4.	Jariyawattanarat et al. (2021) [13]	Fever, Neck swelling with purulent discharge	Long-standing thyroid nodule, iodine deficiency	Streptococcus sui	Incision and drainage, Antibiotics	Survived
5.	Mishra et al. (2021) [14]	Fever, painful dysphagia, drooling of saliva	T2DM, HTN, Hyperlipidemia	Bacteroides fragilis	Antibiotics	Survived
6.	Siti Sanaa binti et al. (2021) [15]	Neck swelling with pain, odynophagia, Fever	Mycobacterium tuberculosis	Streptococcus anginosus and eikenella corrodens	Incision and drainage, Hemithyroidectomy	Survived
7.	Akhanli et al. (2020) [16]	Fever, Dysphagia, Neck swelling with pai, Palpitation	None	Eikenella corrodens	Antibiotics	Survived
8.	Win Htet et al. (2020) [17]	Fever, neck pain, hand tremors,	HTN, Hyperlipidemia	Propionibacterium acnes	Aspiration of abscess, antibiotics	Survived
9.	Khoo et al. (2020) [18]	Neck swelling with pain, Odynophagia, Fever, Dysphagia, odynophagia, hoarseness of voice, neck swelling with pain	T2DM HIV	Salmonella enteritidis	Surgical abscess drainage Hemithyroidectomy	Survived
10	AIYousef et al. (2020) [19]	Neck pain, fever, dysphagia	T2DM, HTN, Hypothyroidism, GERD Hyperlipidemia	Salmonella enterica	Multiple times percutaneous Aspiration Antibiotics	Survived

Differentiating these conditions is extremely important as their treatment differs. While antibiotics with or without surgery are required for patients with acute suppurative thyroiditis, patients with subacute thyroiditis require only analgesics and sometimes corticosteroids. For Emergency physicians, since the results of FNAC are often not available, it is prudent that a low threshold is maintained for initiating antibiotics in patients with painful thyroid swelling since untreated acute suppurative thyroiditis is life-threatening. For serious infections, parenteral antibiotics are necessary. Microscopic examination, staining, and culture of a fine-needle aspirate help determine the best agent. Thionamides are not necessary for individuals with thyrotoxicosis from thyroiditis because there is no excessive thyroid hormone production; nevertheless, adjuvant medications such as beta-blockers and cholestyramine may be used to reduce symptoms or more quickly restore euthyroidism. If a thyroid abscess is found, aspiration or surgical drainage should be tried [20]. Open surgery with a complete, nearly entire, or hemithyroidectomy can be utilized to relieve pressure sensations in extreme circumstances, in individuals who do not respond to suitable antibiotic therapy and drainage. Ultimately, surgical removal of the damaged thyroid lobe may be required to treat the local source of infection and inflammation if systemic indications and symptoms do not improve with initial treatment [21].

Our patient was a young male who was diagnosed case of CKD, who had a mildly painful neck swelling along with symptoms of hyperthyroidism and uremia, and who was ultimately diagnosed to have bacterial suppurative thyroiditis. Although patients with CKD suffer from an altered immune response which includes both innate and acquired immunity [22]. Our patient was on no immunosuppressant drugs. Also, the clinical presentation of our patient was more suggestive of subacute thyroiditis as compared to acute suppurative thyroiditis. Thus, this was a rare presentation of acute suppurative thyroiditis.

## Conclusions

Rare but potentially fatal, acute suppurative thyroiditis should never be ignored. Patients may experience acute symptoms or develop a more chronic course depending on the underlying organism. Its presentation strongly resembles that of subacute thyroiditis, which is a self-remitting illness; precise diagnosis is therefore challenging in the emergency room. Thyroid abscess can have a variety of complications, including thyroid storm, internal jugular vein thrombosis, airway obstruction, tracheal or esophageal perforation, necrotizing mediastinitis, sepsis, and even death. Therefore, all patients who report to the emergency room with painful neck swellings must receive careful attention. The most accurate diagnosis is achieved by fine needle aspiration from thyroid tissue; however, ultrasound is the preferred diagnostic method in the emergency room. Treatment options include intravenous antibiotics, abscess drainage, and possibly surgery.
